# Nephrotic Syndrome as a Cause of Transient Clinical Hypothyroidism

**DOI:** 10.1155/2021/5523929

**Published:** 2021-08-30

**Authors:** Vânia Benido Silva, Maria Teresa Pereira, Carla Leal Moreira, Sílvia Santos Monteiro, Isabel Inácio, Maria Helena Cardoso

**Affiliations:** ^1^Department of Endocrinology, Centro Hospitalar e Universitário do Porto, Porto, Portugal; ^2^Department of Nephrology, Centro Hospitalar e Universitário do Porto, Porto, Portugal; ^3^Department of Endocrinology, Centro Hospitalar do Baixo Vouga, Aveiro, Portugal

## Abstract

Nephrotic syndrome may trigger the onset of hypothyroidism, promoting massive urinary protein losses including thyroxine (T4) and triiodothyronine (T3) along with their binding proteins. At an early stage, a clinical and biochemical euthyroid state is expected. However, in patients with prolonged and severe proteinuria, especially with concomitant low thyroid reserve, urinary losses of free and protein-bound thyroid hormones are sufficiently pronounced to induce a subclinical or overt hypothyroidism. Despite its high prevalence in clinical practice, the literature lacks case reports of newly diagnosed clinical hypothyroidism due to NS in adults, making this condition under-recognized. We report a case of a 23-year-old man with previous normal thyroid function who developed overt hypothyroidism due to a severe nephrotic syndrome, requiring supplementation with levothyroxine (LT). After the patient had undergone bilateral nephrectomy, treatment with LT was discontinued and thyroid function normalized.

## 1. Introduction

The thyroid and kidney interact with each other in a crucial way to assure the normal functioning of both organs. While the thyroid plays a huge role in renal development and growth, as well as in the tubular and glomerular functions, the kidney is involved in the metabolism and elimination of thyroid hormones [1, 2]. The association between thyroid function abnormalities, particularly hypothyroidism, and chronic kidney disease (CKD) is well established and is highly prevalent in clinical practice [3–7]. In this clinical scenario, thyroid function test results are quite variable depending on the renal disease itself and its severity (including progressive impaired kidney function) [4, 5], but also on the patient's previous history of thyroid disease and thyroid reserve [8], as well as on iatrogenic factors (such as the use of glucocorticoids, furosemide, or others) [9].

Nephrotic syndrome (NS) is one of the most common glomerular diseases and is classically characterized by massive proteinuria (>3.5 g/24 hours), hypoalbuminemia, edema, and hyperlipidemia. Associated excessive urinary protein excretion, justified by marked increase in the glomerular permeability to macromolecules, results in urinary losses of thyroid hormone-binding proteins (thyroxine binding globulin (TBG), transthyretin, and albumin), as well as thyroid hormones (thyroxine (T4) and triiodothyronine (T3)) usually bound to them [8, 10]. Additionally, injury of renal tubules can coexist and compromise the reabsorption of free thyroid hormones [11].

In early stages of the disease, especially when there is no prior history of thyroid disorder, levels of physiologically crucial free T3 (fT3) and T4 (fT4) remain normal and a clinical and biochemical euthyroid state is expected. However, in patients with prolonged and severe proteinuria and with low thyroid reserve, urinary losses of free and protein-bound thyroid hormones are sufficiently pronounced to induce an increase in thyroid-stimulating hormone (TSH) values resulting in subclinical or overt hypothyroidism [2, 4, 5]. Dose increase and frequent adjustments may be needed in patients previously treated with hormone replacement therapy [2, 8, 11].

Other conditions may simultaneously contribute to changes in thyroid function laboratory results in patients with NS. When evaluating and managing challenging NS patients, the use of steroids or other immunosuppressant drugs and the euthyroid sick syndrome, a very common entity in hospitalized ill individuals [9, 12], should be considered.

Several studies linking hypothyroidism to NS patients have been published, mostly in children and in patients with preexisting thyroid dysfunction [11, 13–16]. To date, the literature lacks case reports of newly diagnosed clinical hypothyroidism due to NS in adults, remaining as an under-recognized condition.

We report a case of a 23-year-old man with previous normal thyroid function who developed overt hypothyroidism due to a severe nephrotic syndrome, requiring supplementation with levothyroxine (LT). After the patient had undergone bilateral nephrectomy, he started renal function replacement therapy (hemodialysis) and treatment with LT was discontinued and thyroid function normalized.

## 2. Case Report

A 23-year-old man was evaluated by his general practitioner for mild asthenia and generalized edema with swelling of the face, abdomen, and lower limbs over the preceding two weeks. He was an active smoker. His past medical history was positive for antiphospholipid syndrome and Raynaud's phenomenon, and he was on treatment with acenocumarol. The physical examination was unremarkable, except for the presence of an exuberant edema of the legs and abdominal wall. His weight was 78 kg, corresponding to a weight gain of 10% in a 3-month period. No evidence of heart failure was noted.

Urinalysis was positive for proteinuria of 53.73 g/L (reference range (RR): 0.00–0.15 g/L), with a protein-to-creatinine ratio of 13.16 g/g (RR: 0.015–0.068 g/g). Blood tests revealed hypoproteinemia of 4.74 g/dL (RR: 6.0–7.3 g/dL), hypoalbuminemia of 2.56 g/dL (RR: 3.5–5.0 g/dL), and hyperlipidemia (triglycerides: 380 mg/dL (RR: 40–160 mg/dL) and total cholesterol: 377 mg/dL (RR: 0–200 mg/dl)). Liver function tests were normal, and renal depuration was preserved (serum creatinine: 0.79 mg/dL (RR: 0.7–1.2 mg/dL); an estimated glomerular filtration rate of 126.6 mL/min/1.73 m^2^, according CKD-EPI equation) [17]. At this point, the serum TSH level was 2.77 *μ*UI/mL (RR: 0.30–3.18). Renal ultrasound (US) showed preserved anatomy of the kidneys, except for poor corticomedullary differentiation.

The patient was hospitalized for further investigation of newly diagnosed nephrotic syndrome and underwent a renal biopsy, whose result was consistent with C3 glomerulonephritis. A high dose of glucocorticoids was initiated, and due to poor response, a step-up approach was adopted with cyclosporine and mycophenolate mofetil being added to the therapy strategy. Unfortunately, the patient evolved with anasarca, worsening of renal function, severe hypoalbuminemia, and recurrent episodes of cellulitis.

Two weeks after his admission, abnormalities on thyroid function tests were detected (TSH: 17.40 *μ*UI/mL (RR: 0.3–3.18), total T4: 1.8 *μ*g/dL (RR: 5.56–9.91), fT4: 0.33 *μ*g/dL (RR: 1.01–1.65), total T3: 0.4 ng/mL (RR: 0.83–1.49), fT3: 0.81 pg/mL (RR: 2.66–4.33)) accompanied by worsening of hypoalbuminemia to 1.76 g/dL (RR: 3.5–5.0 g/dL) and occasional proteinuria to 59.59 g/L (RR: 0.00–0.15 g/L), prompting further evaluation from Endocrinology Department. The patient had no personal or family history of thyroid disease. On examination, his thyroid was palpable but not enlarged and no nodules were identified. A complementary thyroid study was conducted: serum anti-thyroid peroxidase and anti-thyroglobulin antibodies titers were both negative and thyroid US revealed a slightly enlarged thyroid gland (upper limit of normal dimensions), homogeneous and regular, without evident nodular lesions.

The diagnosis of clinical hypothyroidism secondary to nephrotic syndrome was assumed, although a possible contribution of euthyroid sick syndrome on thyroid hormone levels was considered. The patient was started on oral LT treatment 50 *μ*g daily, with gradual increase in the dose up to 125 *μ*g (1.5 *μ*g of LT per kg of body weight).

**Figure 1:**
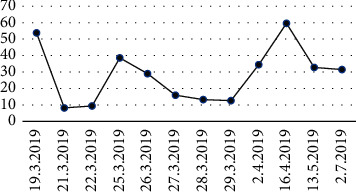
Evolution of proteinuria levels on urinalysis since nephrotic syndrome diagnosis until bilateral nephrectomy.

After three months of hospitalization, his clinical condition remained refractory to the immunosuppressive therapy, with a high variability but progressive worsening of urinary protein levels despite prolonged treatment (Figure 1). Due to unresponsiveness to immunosuppression, steroid side effects, and recurrent severe infections, the patient was proposed to bilateral nephrectomy. Full kidney histology was consistent with focal segmental glomerulosclerosis. Thyroid function reassessment performed six days after surgery revealed that TSH was 2.47 *μ*UI/mL (RR: 0.3–3.18), fT4 was 0.93 *μ*g/dL (RR: 1.01–1.65), and fT3 was 1.67 ng/dL (RR: 2.66–4.33), allowing weaning of LT dose to 75 *μ*g daily.  Table 1 shows the evolution of thyroid function tests over time under different doses of LT, before and after nephrectomy.

After discharge, the patient remained clinically and analytically euthyroid, with progressively lower dose requirements of LT. Eight months after hypothyroidism diagnosis, the serum TSH level was 1.35 *μ*UI/mL (RR: 0.3–3.18) and fT4 was 1.01 *μ*g/dL (RR: 1.01–1.65) on LT 25 *μ*g replacement and therefore, treatment with LT was discontinued. Four months later, in the absence of LT, thyroid function remained normal (TSH: 0.91 *μ*UI/mL (RR: 0.3–3.18); fT4: 1.39 *μ*g/dL (RR: 1.01–1.65)), with a serum albumin level of 3.92 g/dL (RR: 3.5–5.0 g/dL). Since bilateral nephrectomy, the patient was started on renal function replacement therapy (hemodialysis) and is on a waiting list for renal transplantation.

## 3. Discussion

Thyroid hormones, T3 and T4, are both poorly soluble in water, and more than 99.5% circulate in the blood bound to proteins: approximately 70% bound to TBG, 20% to albumin, and 10% to prealbumin. However, only the low levels of free hormones are metabolically active at the tissues and responsible for all thyroid functions [12, 18].

Under normal conditions, urinary protein losses are insignificant. In NS, on the other hand, there is massive urinary protein losses, including T3 and T4 along with their binding proteins and, to a lesser extent, the free fractions fT3 and fT4 [2, 4, 5, 19]. This is responsible for the frequent abnormalities found in thyroid function in patients with NS representing a renal-endocrine disorder [2]. In early stages of the disease or in milder clinical conditions, serum free hormone levels remain normal and the patient is in euthyroid state, as it was visible in our case, when the patient had already-established NS and the serum TSH value was in the normal range [2, 4]. Nevertheless, in more prolonged situations with worsening of proteinuria and hypoalbuminemia, and especially when associated with a low thyroid reserve, fT3 and fT4 are also significantly reduced and overt hypothyroidism may occur [4, 19]. In this case report, with the massive increase in urinary protein losses and the presence of severe and supplemental-dependent hypoalbuminemia, the patient developed clinical hypothyroidism.

The relationship between thyroid dysfunction and the degree of proteinuria, renal function, and serum albumin levels has been documented in several reports. Jain et al. showed that serum TSH levels in NS patients correlated positively with proteinuria and plasmatic creatinine levels but negatively with serum albumin concentrations as well as glomerular filtration rate [4]. In a study with 317 patients who had been definitively diagnosed with NS, it was proved that high levels of urinary proteins and serum creatinine were independent risk factors for predicting thyroid dysfunction, while a higher level of plasmatic albumin was an independent protective factor [5].

Likewise, some published clinical cases have shown that NS may be the cause behind the increased requirements of LT in patients previously diagnosed with hypothyroidism [8, 11, 15, 16].

In this particular case, the patient had no prior history of thyroid dysfunction, he had negative thyroid autoantibodies, and there were no signs of the typical glandular structural changes suggesting diffuse autoimmune thyroiditis and insufficient functional thyroid reserve. The clinical hypothyroidism was triggered by significant urinary losses of hormones and their binding proteins, requiring LT replacement. Furthermore, we speculate that intestinal edema resulting from severe hypoalbuminemia compromised LT absorption with a progressive need for drug titration. As soon as the huge proteinuria levels were eliminated by performing bilateral nephrectomy, an immediate improvement in thyroid function was documented, and after eight months, it was possible to discontinue LT therapy. The permanent resolution of hypothyroidism following bilateral nephrectomy had already been reported in five children diagnosed with congenital NS [14]. This outcome confirms that there was no intrinsic defect in the thyroid gland contributing to hypothyroidism.

However, in this case scenario, we must consider other potential contributors for the thyroid hormonal profile, particularly the administration of high corticosteroid doses over a four-month period of hospitalization. Corticosteroids could promote thyroid dysfunction by their suppressive effect on serum TSH levels, impairment of peripheral conversion of T4 in T3, and the decrease of TBG production [9].

In addition, euthyroid sick syndrome is a very common entity defined by changes in the thyroid function that occur during a period of critical non-thyroid acute or chronic systemic illness. The typical observed hormonal pattern is low T3 levels, eventually low T4 levels, accompanying with reciprocal high reverse T3 and no significant increase in TSH [20]. The magnitude of the changes is proportional to the severity of illness and length of hospital stay [12, 20]. Considering the extremely low concentrations of thyroid hormones that our patient presented, particularly T3, his severe medical condition, and the prolonged hospitalization, his thyroid dysfunction probably results from an overlapping of this syndrome.

In addition to NS, cases of reversible proteinuria and biopsy-proven glomerulonephritis (GN) including membranous nephropathy, minimal change, membranoproliferative GN, amyloidosis, and IgA nephropathy have been reported in association with hypothyroidism, mainly if autoimmune thyroiditis has been present [2, 21]. Several other mechanisms can justify the higher risk for the development of thyroid dysfunction in CKD patients, such as iodine retention and possible Wolff–Chaikoff effect, the reduced deiodinase activity, or the metabolic acidosis frequently present [2, 21, 22].

In summary, we report a case of a 23-year-old patient without previous thyroid disease, who developed severe steroid-resistant NS and clinical hypothyroidism as a consequence of massive urinary protein losses. Bilateral nephrectomy with consequent anuria allowed LT discontinuation.

Renal disease, with or without kidney failure, is a condition that should be considered in the diagnostic workup of newly diagnosed or worsening of preexisting hypothyroidism, especially in hospitalized ill patients when other causes were excluded. Proteinuria severity, drug interactions, and clinical conditions simultaneously determine thyroid dysfunction severity and therapeutic response.

This case report highlights the importance of a greater awareness of the interactions between thyroid and renal functions, often a challenge for clinicians, and that still remains under-recognized, especially in adult patients. It seems reasonable to test thyroid function when nephrotic syndrome is first diagnosed and then perform serial testing.

## Figures and Tables

**Table 1:** Description of evolution of thyroid function tests over time under different doses of LT, before and after nephrectomy.

Date	15.3.2019	8.4.2019	16.4.2019	29.4.2019	13.5.2019	26.6.2019	∗8.7.2019	11.9.2019	20.03.2020
LT dose/day	—	—	50 *μ*g	100 *μ*g	112 *μ*g	125 *μ*g	125 *μ*g	75 *μ*g	—
TSH (*μ*UI/mL) (RR: 0.3–3.18)	2.77	17.40	11.60	7.94	2.66	1.71	2.47	0.15	0.91
fT4 (*μ*g/dL) (RR: 1.01–1.65)		0.33	0.74	0.71	0.77	0.65	0.93	1.29	1.39
fT3 (pg/mL) (RR: 2.66–4.33)		0.81	0.84	0.39	0.53		1.67	2.74	
Total T4 (*μ*g/dL) (RR: 5.56–9.91)		1.8	1.8					5.4	
Total T3 (ng/mL) (RR: 0.83–1.49)		0.4	0.3					0.8	

^*∗*^Bilateral nephrectomy (2.7.2019).

## References

[1] Bradley S. E., Stéphan F., Coelho J. B., Réville P. (1974). The thyroid and the kidney. *Kidney International*.

[2] Dousdampanis P., Trigka K., Vagenakis G. A., Fourtounas C. (2014). The thyroid and the kidney: a complex interplay in health and disease. *The International Journal of Artificial Organs*.

[3] Chonchol M., Lippi G., Salvagno G., Zoppini G., Muggeo M., Targher G. (2008). Prevalence of subclinical hypothyroidism in patients with chronic kidney disease. *Clinical Journal of the American Society of Nephrology*.

[4] Jain D., Aggarwal H. K., Pavan Kumar Y. M., Jain P. (2019). Evaluation of thyroid dysfunction in patients with nephrotic syndrome. *Medicine and pharmacy reports*.

[5] Li L.-Z., Hu Y., Ai S.-L. (2019). The relationship between thyroid dysfunction and nephrotic syndrome: a clinicopathological study. *Scientific Reports*.

[6] Lo J. C., Chertow G. M., Go A. S., Hsu C.-Y. (2005). Increased prevalence of subclinical and clinical hypothyroidism in persons with chronic kidney disease. *Kidney International*.

[7] Rhee C. M., Kalantar-Zadeh K., Streja E. (2015). The relationship between thyroid function and estimated glomerular filtration rate in patients with chronic kidney disease. *Nephrology Dialysis Transplantation*.

[8] Chandurkar V., Shik J., Randell E. (2008). Exacerbation of underlying hypothyroidism caused by proteinuria and induction of urinary thyroxine loss: case report and subsequent investigation. *Endocrine Practice*.

[9] Burch H. B. (2019). Drug effects on the thyroid. *New England Journal of Medicine*.

[10] Fonseca V., Thomas M., Katrak A., Sweny P., Moorhead J. F. (1991). Can urinary thyroid hormone loss cause hypothyroidism?. *The Lancet*.

[11] Jung S. H., Lee J. E., Chung W. Y. (2019). Changes in the thyroid hormone profiles in children with nephrotic syndrome. *Korean Journal of Pediatrics*.

[12] Maiden M. J., Torpy D. J. (2019). Thyroid hormones in critical illness. *Critical Care Clinics*.

[13] Benvenga S., Vita R., Di Bari F., Fallahi P., Antonelli A. (2015). Do not forget nephrotic syndrome as a cause of increased requirement of levothyroxine replacement therapy. *European Thyroid Journal*.

[14] Chadha V., Alon U. S. (1999). Bilateral nephrectomy reverses hypothyroidism in congenital nephrotic syndrome. *Pediatric Nephrology*.

[15] Karethimmaiah H., Sarathi V. (2016). Nephrotic syndrome increases the need for levothyroxine replacement in patients with hypothyroidism. *Journal of Clinical and Diagnostic Research: JCDR*.

[16] Soh S., Aki O., Manabu O., Norimasa K., Hiroshi K., Masao N. (2016). A case of minimal change nephrotic syndrome with hypothyroidism deterioration. *CEN Case Reports*.

[17] Levey A. S., Stevens L. A., Schmid C. H. (2009). A new equation to estimate glomerular filtration rate. *Annals of Internal Medicine*.

[18] Bartalena L., Robbins J. (1993). Thyroid hormone transport proteins. *Clinics in Laboratory Medicine*.

[19] Alam A. B. M., Hasan A. N. M., Khan A. H., Quader M. M. U., Begum S. A., Banik S. K. (2013). Association between primary hypothyroidism and nephrotic syndrome: a case report. *Chattagram Maa-o-Shishu Hospital Medical College Journal*.

[20] Lee S., Farwell A. P. (2016). Euthyroid sick syndrome. *Comprehensive Physiology*.

[21] Mariani L. H., Berns J. S. (2012). The renal manifestations of thyroid disease. *Journal of the American Society of Nephrology*.

[22] Connie M. R. (2016). The interaction between thyroid and kidney disease: an overview of the evidence. *Current Opinion in Endocrinology, Diabetes & Obesity*.

